# Technology facilitated sexual violence: a comparative study between working and non-working females in Egypt before and during the COVID-19 pandemic

**DOI:** 10.1186/s41935-022-00278-2

**Published:** 2022-04-11

**Authors:** Nancy M. Zagloul, Rasha M. Farghaly, Hossam ELKhatib, Sahar Y. Issa, Safaa M. El-Zoghby

**Affiliations:** 1 Department of Forensic Medicine and Toxicology, Faculty of Medicine, Misr University for Science and Technology, October City, Egypt; 2 Department of Community, Occupational and Environmental Medicine, Faculty of Medicine, Suez Canal University, Ismailia, Egypt; 3 Department of Psychiatry, Faculty of Medicine, Misr University for Science and Technology, October City, Egypt; 4 Department of Forensic Medicine and Clinical Toxicology, Faculty of Medicine, Alexandria University, Alexandria, Egypt; 5 Department of Family Medicine, Faculty of Medicine, Suez Canal University, Ismailia, Egypt

**Keywords:** Digital violence, Sexual violence, Technology facilitated sexual violence, COVID-19, Online harassment, Egypt

## Abstract

**Background:**

During the COVID-19 pandemic, quarantine measures policies increased Internet usage, leading to technological hazards as technology facilitated sexual violence (TFSV).

**Aim:**

The current work aimed to assess TFSV among working and non-working Egyptian females before and during COVID-19.

**Methods:**

The current work is a cross-sectional observational comparative study using an anonymous online questionnaire distributed through social platforms among working and non-working Egyptian females.

**Results:**

TFSV was reported by 50.3% of the participants; however, regarding some forms of digital sexual violence, there was a significant decrease during COVID-19 lockdown than before it, considering; threatened creation form (7.8%, 12.0%; *p* = 0.017); non-consensual pornography (31.4%, 51.9%; *p* < 0.001) and online sexual harassment and cyber-stalking types (80.9%, 89.4%; *p* < 0.001). Only 17.3% of the study participants knew the identity of the perpetrator. TFSV led 6.4% to abstain from social media, and 3.9% reported the incident to a law agency.

**Conclusions:**

The current study revealed that almost half of women experienced TFSV. Although time spent on the Internet by the whole participants during the pandemic was significantly higher than before, there was a significant decrease in some types of TFSV. The current study revealed that divorced females working in non-governmental sectors experienced harassment more significantly than others. There is crucial importance to set laws and penalties against perpetrators of TFSV to provide a safe technological environment for women.

## Background

Cyber violence is a new type of interpersonal violence brought on by modern technology. Although the scientific community has produced various instruments to measure this phenomenon, there have been no systematic evaluations that compare measurements focusing on different population groups in recent years. In romantic relationships, digital media has arisen as a new route for engaging in and experiencing violent behaviors. According to a recent study, this type of dating violence has been labeled as electronic dating violence, cyber aggression, online dating abuse, cyber violence, and cyber dating abuse, among other terms (Rodríguez-deArriba et al. 2021).

The recent surge in technology, including social networking sites, personal blogs, video sharing, and cellphones in the present era of modernism and globalization, is a developing element of contemporary society. This technology advancement helped evolve a new type of violence termed cyber violence (Henry and Powell 2014).

United Nations Office on Drugs and Crime (UNODC) reported that the COVID-19 quarantine measures and policies of self-isolation had increased Internet use by 50 to70%, and everyone resorted to the web for work, schooling, and social activities (UNODC 2020). Greater use during lockdowns, with increased boredom and concern for sexual thinking, led to increased harassment and online violence (Parks et al. 2020).

Sexual harassment (SH) is not a new issue, but the fast-evolving digital technology has introduced new and rapid developments and complex challenges conveyed through digital methods. In recent decades, SH has been thoroughly investigated in terms of prevalence, outcomes, and prevention; many studies provide an interdisciplinary perspective covering the phenomenon's psychological, sociological, medical, legal, and educational aspects. SH potentially relates to any human being; however, most victims are women (Mitchell et al. 2014; Henry and Powell 2018; Snaychuk and O'Neill 2020).

TFSV consists of six distinct but related categories (Cripps 2016). One of these categories is the unauthorized fabrication or distribution of sexually explicit images of non-consensual pornography or revenge porn victims on cyberspaces.

The Internet provides an environment where healthy and pathological behaviors may be practised. Technology advancements increased verbal or graphical sexual violence that expanded with websites built expressly to post naked photographs of people and identify information such as social media profiles and full or partial names (Stroud 2014; Henry and Powell 2015a).

Another category of TFSV involves the fabrication or distribution of images of sexual assault, while the third form of TFSV includes carriage services to ensure a sexual assault. This includes publishing an Internet advertisement inciting someone to assault another person in vengeance. The fourth kind of TFSV comprises both cyber sexual harassment and stalking. This category covers actively harassed forms such as sex and degrading comments on Internet forums and chat rooms. Online sexual harassment might involve requesting or sending unsolicited pornography to someone’s queries via the Internet. This category also covers passive harassing methods such as sexually evocative surnames (Barak 2005).

Finn and Banach (2000) considered page jacking a form of online sexual harassment. This is encountered when an Internet user hijacks a webpage and redirects site visitors to a website containing pornographic or other offensive material. In contrast, cyberstalking means a person repeatedly pursuing electrical or Internet-enabled gadgets (Reyns et al. 2012). Such repeated actions include unwanted electronic messages that can be menacing, frightening, or coercive (Hazelwood and Koon-Magnin 2013).

The fifth form of TFSV is gender-based hate speech, which includes rude and demeaning statements addressed at an individual or a gender-based group, usually women. The last form of TFSV is virtual rape, when a person’s avatar (digital representative of people) is subjected to simulated sexual violence by other avatars (Henry and Powell 2015b).

Women and girls often used the Internet during the pandemic, as there is a digital divide between the sexes. While there is still a lack of a complete worldwide definition of information and communication technology (ICT) statistics facilitating violence, research reveals that women are overly targeted and have serious repercussions (OHCHR 2018). As the pandemic of COVID-19 worsens economic and social hardship and restricts movement and social isolation, sexual violence escalates rapidly (UNWomen 2020b).

TFSV is a growing public and social-health problem, and although being a prevalent phenomenon, it is still understudied, with initial research investigations demonstrating significant adverse consequences. Only a few Egyptian studies assessed and documented digital sexual harassment, especially during the COVID-19 pandemic (Arafa and Senosy 2017; Arafa et al. 2018; Hassan et al. 2020). This work was conducted to determine TFSV among working and non-working Egyptian females before and during the pandemic of COVID-19.

## Methods

### Study design and population

Current research consists of a cross-sectional observational study to evaluate online sexual harassment of working and non-working Egyptian women through an anonymous online questionnaire during the COVID-19 pandemic. The survey was distributed using a link shared via emails or social networking sites between 15 and 30 July and 2020. Google forms with an online semi-structured questionnaire, including a consent form. We received responses from 283 adult females only who met the criteria for inclusion and agreed to share their results with convenient sampling strategies for snowballing (Naing 2003; Henry and Powell 2015a).

### Inclusion and exclusion criteria

Adult women aged 18 and above living in Egypt, who have understood and read Arabic, have used social media, and are prepared to provide informed permission were included in the current study. The study excluded teens and non-Egypt inhabitants.

### Study tools

Online semi-structured modified, and validated questionnaire consisted of four parts (Finn 2004; Foundation, D. R. 2017; Snaychuk and O'Neill 2020):

1. Sociodemographic characteristics: age, nationality, occupation, the use of the Internet and technology in work, education, and social status.
2. Internet usage: including items such as hours per day using the Internet, the used devices, and amount of personal information (e.g., photos, details of private life) that exists online.
3. Online sexual harassment: this part investigated the females’ perception about online sexual harassment definition, if they were being harassed, the frequency of the harassment, and its types, before and during the COVID-19 pandemic.
4. Perpetrator and effect on using the Internet: these are four questions asked about the identity of the perpetrator(s), his relationship to the victim, and the actions taken after being harassed; whether abstain from social media use or report the incidence to any law agency.

### Validation and pilot study

Pilot research with an acceptable Cronbach’s α of 80% was conducted on 10 participants before the study to examine the feasibility and reliability of the questionnaire. The questionnaire was designed in Arabic and English. Three experts validated the questionnaire.

### Data collection

Data were gathered using a Google form with an online semi-structured questionnaire, including a consent statement to approve participation in the study. The survey link was delivered via emails, WhatsApp™, Facebook™ groups, and other social media sites. The participants were asked to send the survey link as far as possible to other people. Once the study was received and the participants clicked on the link, they were informed and consented. They filled up the demographic characteristics once they agreed to complete the poll. Then, several questions appeared consecutively and had to be answered.

### Outcome variables

Studying the prevalence of TFSV among Egyptian females, comparing digital sexual harassment before and during the COVID-19 pandemic, comparing digital sexual harassment in working and non-working females before and during COVID-19.

### Statistical analysis

Statistical analysis was carried out using the Social Sciences Statistical Package (SPSS V23.0). Using the Shapiro-Wilk test, the normal distribution of the continuous variables was tested. Average and standard deviation were used for descriptive statistics of quantitative data. Frequencies and percentages for qualitative variables were calculated and tabulated. Inferential statistics were utilized for quantitative and qualitative data testing by Mann-Whitney, Wilcoxon signed ranks, and McNemar, and logistic regression analysis was performed. *P* values < 0.05 were adopted as a significance threshold.

### Research ethics

The Ethics Committee of the Suez Canal and Alexandria Universities Medical Faculties approved the study. Informed permissions were obtained from all subjects participating in the survey. There were no monetary rewards to complete the questionnaire.

## Results

The current study was conducted on 563 Egyptian females, of whom 283 (50.3%) participants reported being exposed to cyber violence. The mean age of the participants (29.9 ± 8.5) ranged between 18 and 60 years. Most of the participants were Egyptian (96.1%). Working and non-working females were almost equally represented (55.5% and 44.5%, respectively), the mean duration of work is 11.3 ± 6.7 ranging from 0.2 to 30 years. Concerning living conditions, 50.2% lived with their families (father and/or mother while only 4.9% lived alone. Participants with a university level of education conferred 64.3% of the study sample. Single participants represented 48.8% of the study sample regarding social status, while only 0.2% were widows (Table 1).

**Table 1:** Sociodemographic data of the harassed study participants (*n* = 283)

Sociodemographic data	No.	%
Age (in years) (mean ± SD, range)	29.9 ± 8.5	18–60
**Nationality**		
Egyptian	272	96.1
Non-Egyptian	11	3.9
**Working status**		
Working	157	55.5
Not working	126	44.5
**Type of working institute**		
Governmental	89	31.4
Private	58	20.5
Self-employed	10	3.5
**Duration of work (in years) (mean ± SD, range)**	11.3±6.7	0.2-30
**Living conditions**		
Family (mother and/or father)	142	50.2
Roommates	15	5.3
Husband and kids	112	39.6
Alone	14	4.9
**Education**		
Primary	2	0.7
Secondary	3	1.1
University	182	64.3
Postgraduate	96	33.9
**Social status**		
Single	138	48.8
In relationship/dating	25	8.8
Married	108	38.2
Divorced	10	3.5
Widow	2	0.7
**Socioeonomic status**		
Upper class	40	14.1
Upper middle class	59	20.9
Middle class	117	41.3
Upper lower class	22	7.8
Lower class	45	15.9

**Fig. 1:**
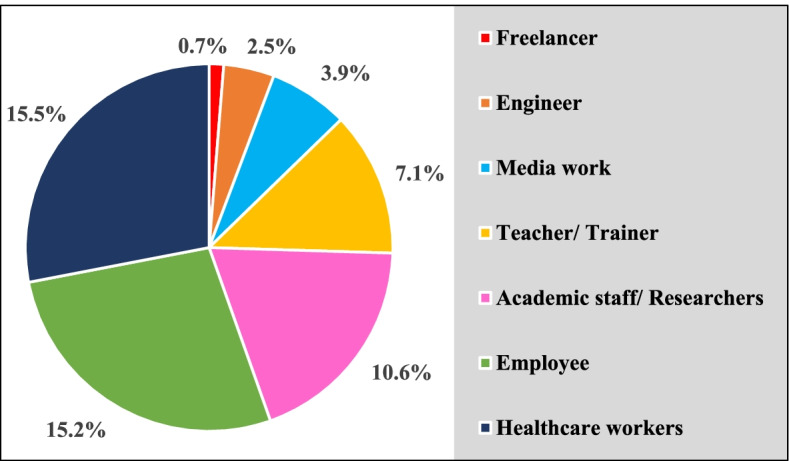
Occupations of the study participants (*n* = 283)

As shown in Fig. 1, the highest represented occupations among the study participants were healthcare workers (15.5%), employees (15.2%), and academic staff/ researchers (10.6%). Table 2 shows that the most used devices for the Internet were smartphones (98.2%). Only 10.2% of the participants had much online personal information, while 41.7% and 35.3% had little and some information, respectively. The mean hours spent on the Internet were significantly higher during COVID-19 lockdown (7.6 ± 4.0) than before lockdown (4.7 ± 3.0) (*P* value < 0.001), where hours spent on the Internet were substantially higher among working than non-working both before and during COVID-19 lockdown as presented in Table 3.

**Table 2:** Internet usage among the harassed study participants (*n* = 283)

Internet usage	Number	%	
The device used every day (number, %)			
Smartphone	278	98.2	
Tablet/iPad	16	5.7	
Computers/laptop	88	31.1	
Amount of personal information (photos, details of personal life) exists online (number, %)			
None	36	12.7	
A little (< 3)	118	41.7	
Some (3–5)	100	35.3	
A lot (> 5)	29	10.2	
Hours spent on Internet before COVID-19 lockdown (mean ± SD, range)	4.7 ± 3.0	0.5–16	*P* value < 0.001*^§^
Hours spent on Internet during COVID-19 lockdown (mean ± SD, range)	7.6 ± 4.0	0.5–18	

*Statistically significant at *p* < 0.05

^§^Wilcoxon signed ranks test

**Table 3:** Relation between working status and internet usage among the harassed study participants (*n* = 283)

Hours spent on the Internet	Working status		***P*** value^**¶**^
	Working	Not working	
**Before COVID-19 lockdown**	4.5 ± 3.1	4.9 ± 2.3	0.047*
**During COVID-19 lockdown**	7.2 ± 4.1	8.0 ± 4.0	0.042*
***P*** **value** ^**§**^	< 0.001*	< 0.001*	

*Statistically significant at *p* < 0.05

^¶^Mann-Whitney test

^§^Wilcoxon signed ranks test

**Fig. 2:**
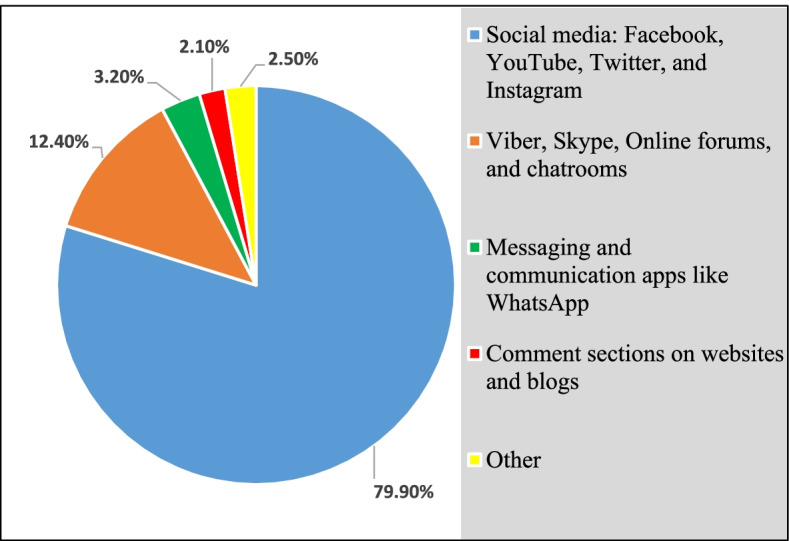
Worst platforms regarding online TFSV as reported by the harassed study participants (*n* = 283)

“Misusing someone’s online data including photos and posts” was the most common definition of online sexual harassment reported by the study participants (74.9%). Besides, social media (Facebook, YouTube, Twitter, Instagram) were the worst platforms regarding online sexual harassment, as reported by 79.9% of the participants, as shown in Table 4 and Fig. 2.

**Table 4:** Attitudes towards digital harassment among the harassed study participants (*n* = 283)

Digital harassments and worst online platforms among participants	Number	%
**Definition of online sexual harassment**		
Misusing someone’s online data, including photos and posts.	212	74.9
Calling someone offensive and abusive things in the comments sections of Facebook posts or websites	180	63.6
Getting into contact with someone’s family or friends and disclosing their personal photos and information	143	50.0
Threatening someone with physical violence for their views	126	44.5
Accessing someone's email or online accounts without their permission	159	56.2
**Worst platforms regarding online sexual harassment**		
Social media: Facebook, YouTube, Twitter, and Instagram	226	79.9
Viber, Skype, Online forums, and chatrooms	35	12.4
Messaging and communication apps like WhatsApp	9	3.2
Comments section on websites and blogs	6	2.1
Others	7	2.5

**Fig. 3:**
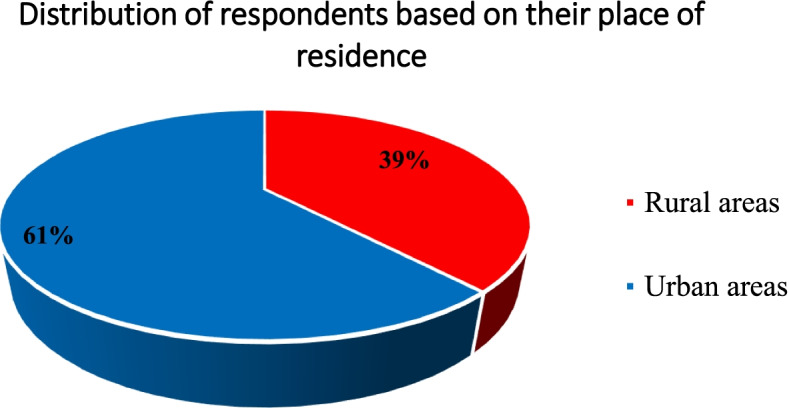
Prevalence of TFSV among the harassed study participants based on their place of residence (*n* = 283)

Figure 3 demonstrated that 61.4% of females who reported digital harassment were from urban areas, and 38.6% were from rural areas. 49.8% of the study participants reported digital harassment. Table 5 outlined a significant decrease during COVID-19 lockdown than before lockdown considering; threatened creation (7.8%, 12.0%; *p* = 0.017); non-consensual pornography (31.4%, 51.9%; *p* < 0.001) and online sexual harassment and cyber-stalking types (80.9%, 89.4%; *p* < 0.001). The incidence of virtual rape, procuring sexual assault, and gender-based hate speech was nearly the same before and during the pandemic.

**Table 5:** Different types of TFSV before and during COVID-19 lockdown as reported by the harassed study participants (*n* = 283)

Type of digital harassment	Before	During	***P*** value^**§**^
Threatened creation	34(12.0%)	22(7.8%)	0.017*
Virtual rape	19(6.7%)	19(6.7%)	1.000
Carriage service to procure a sexual assault before	54(19.1%)	54(19.1%)	1.000
Gender-based hate speech	94(33.2%)	92(32.5%)	0.832
Non-consensual pornography	147(51.9%)	89(31.4%)	< 0.001*
Online sexual harassment and cyber-stalking	253(89.4%)	229(80.9%)	< 0.001*

*Statistically significant at *p* value < 0.05

^**§**^McNemar test

Only 17.3% of the harassed study participants knew the perpetrator's identity, most of whom were friends of friends (4.6%). TFSV led 6.4% to abstain from social media, and 3.9% reported the incident to a law agency (Table 6).

**Table 6:** Perpetrator and effect of digital harassment on using the Internet as reported by the harassed study participants (*n* = 283)

Perpetrator and effect on using Internet	Number	%
**Known identity of the perpetrator**		
Known	49	17.3
Unknown	234	82.7
**Perpetrator identity**		
A Facebook page	1	0.4
Stepfather	1	0.4
Dorm mate	2	0.7
Virtual friend	2	0.7
Other	2	0.7
School mate	3	1.1
Neighbors	3	1.1
First degree relative	3	1.1
Boss	4	1.4
Romantic partner	6	2.1
Friend	7	2.5
Family member	8	2.8
Co-worker	10	3.5
Acquaintance	11	3.9
Friends of friends	13	4.6
**Abstaining social media due to digital harassment**	18	6.4
**Reporting to any law agency**	11	3.9

**Fig. 4:**
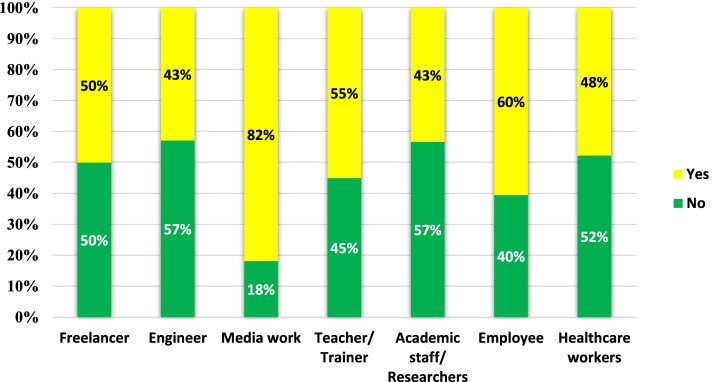
Relation between digital harassment and occupation

As presented in Table 7, harassment showed significant relation with living conditions where 80% of those living with roommates and 71.4% of those living alone were digitally harassed (*p* value = 0.025). Besides, there was a significant relation between digital harassment and social status where 56% of those in relationship/dating and 90% of divorced participants were harassed (*p* value = 0.022). The results also illustrated that those working in media (advertising and photographing) were the most subjected to digital harassment, as shown in Fig. 4. Binary logistic regression analysis was performed to detect the predictors of TFSV using the backward conditional method. Factors entered into the model were age (young age versus middle-aged and elderly), social status (divorced versus other statuses), educational level, work status (employed\unemployed), type of workplace (non-governmental versus governmental workplace), and time spent online. Predictors of TSV as detected by regression analysis were being employed, divorced social status and working in non-governmental workplaces, as presented in Table 8.

**Table 7:** Relation between digital harassment and sociodemographic characteristics of the harassed study participants (*n* = 283)

	Harassed	Not harassed	*P* value
Age (in years) (mean ± SD)	29.9 ± 8.5	29.8 ± 8.6	0.859^€^
Working status			
Working	84(53.5%)	73(46.5%)	0.167^¶^
Not working	57(45.2%)	69(54.8%)	
Type of working institute			
Governmental	42(47.2%)	47(52.8%)	0.088^¶^
Private	34(58.6%)	24(41.4%)	
Self employed	8(80.0%)	2(20.0%)	
Duration of work (in years) (mean ± SD)	11.4 ± 7.0	11.2 ± 6.4	0.947^€^
Living conditions			
Family mother and father	69(48.6%)	73(51.4%)	0.025*
Room matehs	12(80.0%)	3(20.0%)	
Husband and kids	50(44.6%)	62(55.4%)	
Alone	10(71.4%)	4(28.6%)	
Education			
Primary	2(100.0%)	0(0.0%)	0.577^¶^
Secondary	2(66.7%)	1(33.3%)	
University	91(50.0%)	91(50.0%)	
Postgraduate	46(47.9%)	50(52.1%)	
Social status			
Single	71(51.4%)	67(48.6%)	0.022*^¶^
In relationship/dating	14(56.0%)	11(44.0%)	
Married	47(43.5%)	61(56.5%)	
Divorced	9(90.0%)	1(10.0%)	
Widow	0(0.0%)	2(100.0%)	
Hours spent on the Internet			
Before COVID-19 lockdown (mean ± SD)	4.8 ± 3.1	4.6 ± 2.9	0.844^€^
During COVID-19 lockdown (mean ± SD)	8.1 ± 4.2	7.1 ± 3.7	0.077^€^
The amount of personal information (photos, details of personal life) that exists online			
None	14(38.9%)	22(61.1%)	0.538^¶^
A little	59(50.0%)	59(50.0%)	
Some	53(53.0%)	47(47.0%)	
A lot	15(51.7%)	14(48.3%)	

* Statistically significant at *p* value<0.05

^€^ Mann-Whitney test

^¶^ Chi-square test

**Table 8:** Backward logistic regression analysis of predictors of harassment among the study participants

	B	S.E.	*P* value	OR	95% C.I. for OR	
					Lower	Upper
Employed work status	0.664	0.308	0.031*	1.943	1.062	3.555
Divorced social status	2.414	1.073	0.024*	11.178	1.366	91.477
Non-governmental workplace	0.740	0.335	0.027*	2.096	1.086	4.044
Constant	− 0.945	0.381	0.013*	0.389		

*Statistically significant at *p* value < 0.05

## Discussion

Cyber-violence aggressors usually keep a safe distance from their targets, avoiding retaliation. The Internet gives the perfect platform for someone to inflict maximum psychological harm on another at the lowest personal risk; therefore, the Internet is an ideal environment for indirect aggression (Cho and DioGuardi 2020). The current work investigated TFSV and its related factors among Egyptian females before and during the COVID-19 pandemic. Because of the lockdown state that required users to use more Internet services for more hours, authors expected that harassment during a pandemic would grow. This is the first study to explore how the current COVID-19 pandemic affected digital harassment to the best of our knowledge.

The rising usage of social media platforms has resulted in new online aggression and violence forms. According to some studies, cyberbullying and harassment, such as threatening or sexual messages conveyed via social media, are more frequent among most populations (Peterson and Densley, 2017; Liu et al. 2019). The scientific fields of criminology, psychology, and sociology, all concerned with violent conduct, have produced very little research on the prevalence of causation of various forms of cyber violence (Brown 2015).

The current study revealed that almost half (*n* = 283, 50.3%) of the female participants were subjected to digital harassment. This result is slightly close to that found by Hassan et al. in Egypt (41.6%) (Hassan et al. 2020) and by the Hamara Study (40%) in Pakistan (Foundation, D. R. 2017). However, it is far greater than what women in the European Union (Rights, E. U. A. f. F 2014) and Canada (Canada 2019) claim (10% and 20% respectively).

Our participants reported smartphones as the most used device for Internet access (98.2), which is greater than previous studies performed in Canada (72.4%) (Snaychuk and O'Neill 2020) and Pakistan (48%). (Foundation, D. R. 2017) This result may explain the higher levels of reported harassment than the other studies, as smartphones are easily portable devices allowing for more Internet access anywhere and anytime, together with the many social media websites and communication apps that could be downloaded regularly than other devices (Oksman and Turtiainen 2004).

It was revealed in our study that working women spent much more hours on the Internet during the COVID-19 epidemic than before (*P* < 0.001), and working women spent much more hours on the Internet than non-working women before and during the pandemic (*P* = 0.047 and 0.042, respectively). This finding accords with what was proclaimed by the United Nations (UN); that millions of females had to use video conferencing frequently, or even daily, for their work purposes (UN Women 2020b). The pandemic and lockdown measures have increased Internet access between 50% and 70% for work, education, and social activities, whereas those lacking digital skills are more prone to cyber violence. (UN Women 2020a).

In the current research, the females’ perception for the definition of digital harassment, misusing someone’s online data, including photos and posts, was the most popular reply by the majority (about 75%), while calling someone offensive and abusive things in the comments sections of Facebook posts or websites was the least one; accounting for 44.5%. Responding to other forms of harassment varies from 50% to about 64% by the studied population.

This significant variation in perceiving digital harassment is in line with the previous studies in the USA (Duggan 2017) and Pakistan (Foundation, D. R. 2017), showing that females in different countries and with different cultures lack the knowledge and awareness regarding this issue. They might realize many improper online behaviors as acceptable because they are abundant in their communities, which need deep investigation and intervention.

Most of our participants (about 80%) reported social media (e.g., Facebook, Twitter, and Instagram) as the platform to become prone to digital harassment. While this result conforms with that reported previously in Egypt, about three-quarters of the females stated social media as the most common platform for digital harassment (Hassan et al. 2020). This finding is considerably more significant than Pakistan’s results for the same media platforms (50%) (Foundation, D. R. 2017).

Despite the inconsistent results in these different studies, the participants agreed upon considering social media as the worst platform, subjecting them to digital harassment. This result is incongruent with what was mentioned in the Pew research 2017 that social media seemed to be a fertile ground for digital harassment (Duggan 2017). Consistent with earlier studies in the USA (Duggan 2017; Finn 2004) and Egypt (Hassan et al. 2020), the perpetrator was unknown to most of our participants, a matter mainly facilitated by the anonymity nature allowed by the Internet.

On the other hand, only 3.9% of the harassed females in our study reported the action to any law agency, a result that adheres to what mentioned in the Pakistani and UN studies that most of the females have a sense of fragility in being capable, or even have the knowledge, of reacting to the harassment (Foundation, D. R. 2017; UN Women 2020a). Other reasons might be attributed to the judgmental nature of the community that could blame the harassed female rather than the perpetrator and the fear that reporting the incident might smudge their reputation.

The current work revealed that online sexual harassment and cyber-stalking were the most common forms of digital harassment stated by females, whether before or after the COVID-19 pandemic (89.4% and 80.9%, respectively). Likewise, other studies conducted among Egyptian and Canadian females before the pandemic revealed the same result of being the most frequently experienced form of digital harassment (Hassan et al. 2020; Snaychuk and O'Neill 2020).

On comparing different forms of harassment before and during the pandemic, surprisingly, there was a statistically significant reduction in some forms of harassment. Considering the increased Internet access during the COVID-19 pandemic, this was unexpected. This might be explained by religious fear, and the sense of impending death as nearly every family had heard about a fatal outcome of a known COVID-19 infection case, or even due to financial difficulties that directed their scope of using the Internet to seek work not to spend time only as they used to do before the COVID-19 pandemic. These findings were in accordance with the results reported by Rigoli F. in his work studying the link between the COVID-19 pandemic and religious beliefs (Rigoli 2020). COVID-19 pandemic is a major global stressful condition that affects the vast majority of the human population. Research has observed that, when experiencing distressing situations, some people manifest increased religious conviction, and many studies have raised the possibility that increasing religious commitment represents a strategy to cope with stress (Hart and Koenig 2020).

Hawdon et al. reported that cyber victimization is more dependent on the type and cyberspace of online activities that allow access to what they call “dangerous virtual spaces.” This increase can be caused by more insecure online spaces, not online time (Hawdon et al. 2020). Another explanation is that persons who have previously been harassed frequently take active precautions to protect themselves against further online harassment, and they leave the cyberspaces where they were exposed to cyberbullying and harassment (Duggan 2017).

On investigating the relationship between sociodemographic characteristics and digital harassment, the mean age for harassment was about 29 years. Similar results were detected in Europe and the USA, where the risk of victimization was highest among young females aged between 18 and 29 years (Duggan 2017; UN Women 2020a). The current study revealed that divorced females working in non-governmental sectors experienced harassment more significantly than others. On the other hand, nationality, education, and time spent on the Internet had no significant relation to digital harassment. Hassan et al. further showed that neither education, employment, nor the daily use of the Internet had any significance in cyber harassment (Hassan et al. 2020). However, this was in discordance with others that time spent on the Internet was positively associated with more exposure to online harassment (Aletky 2001; Lindsay and Krysik 2012; Nasi et al. 2014; and Arafa and Senosy 2017). These irreconcilable findings may be related to the different age groups of the study population, as most of these studies were on university students where most of their Internet activities are on social media sites that appeared to be the most platform prone to harassment.

## Conclusions

This investigation is an essential step in deciphering the nature and incidence of TFSV in Egypt. Nearly half of the study participating females had experienced TFSV victimization. It was also revealed that the amount of time the participants spent on the Internet during the COVID-19 outbreak was substantially higher than before. Social media were the standard platforms for digital sexual violence (e.g., Facebook, Twitter, and Instagram). Surprisingly, some forms of TFSV decreased after the COVID-19 pandemic, with a statistically significant reduction like non-consensual pornography and Online sexual harassment, and cyber-stalking. TFSV was more encountered among media workers more than all other reported professions. The current study also revealed that divorced females working in non-governmental sectors experienced harassment more significantly than others.

## Recommendation

It is recommended that a comprehensive definition of harmful “online sexual violence” should be reached to implement better legal policy and preventative measures. Currently, this research has aimed to address most forms of TFSV collectively, so further studies are needed to focus on each category of TFSV separately and make distinctions between the different types. Governments and lawmaking initiatives must start adapting to the changing technological nature of crimes against women. Also, setting laws and penalties against perpetrators is crucial to providing a safe technical environment for working or non-working.

## Data Availability

All datasets generated and analyzed during the current study and research materials are available from the corresponding author on request. All authors agree upon the availability of data and materials.

## Abbreviations

TFSV: Technology facilitated sexual violence
UNODC: United Nations Office on Drugs and Crime
ICT: Information and communication technology
UN: United Nations
